# STAT3 down regulates LC3 to inhibit autophagy and pancreatic cancer cell growth

**DOI:** 10.18632/oncotarget.1810

**Published:** 2014-03-15

**Authors:** Jingjing Gong, Amanda R. Muñoz, Daniel Chan, Rita Ghosh, Addanki P. Kumar

**Affiliations:** ^1^ Department of Urology, The University of Texas Health Science Center, San Antonio, TX; ^2^ Division of Medical Oncology, University of Colorado, Aurora, CO; ^3^ Cancer Therapy and Research Center, The University of Texas Health Science Center, San Antonio, TX; ^4^ South Texas Veterans Health Care System, The University of Texas Health Science Center, San Antonio, TX

**Keywords:** Pancreatic cancer, Nexrutine®, STAT3, inflammation, autophagy, LC3

## Abstract

The dismal 5-year survival (<5%) for pancreatic cancer (PanCA) underscores the need for developing effective therapeutic options. Recent studies from our laboratory have shown that Nexrutine^®^ (Nx), a bark extract from *Phellodendron amurense* exhibits excellent anticancer activity in human pancreatic cancer cells through inhibition of inflammatory signaling via STAT3/NFκB/Cox-2. Given the apparent high oxidative stress and autophagic activity in pancreatic tumors, we investigated the potential of Nx to modulate autophagy, reactive oxygen species (ROS), and their crosstalk. Our results show that Nx inhibits autophagy and decreases ROS generation. Pharmacological inhibition of autophagy led to decreased ROS generation and proliferation with no significant effect on apoptosis. Further, using combination index analysis we also found that combination of late-stage autophagy inhibitor with Nx exhibited a moderate synergistic to additive effect. Additionally, genetic or pharmacological inactivation of STAT3 reduced LC3-II levels and expression indicating a possible role for STAT3 in transcriptional regulation of autophagy. Since both inflammatory and oxidative stress signaling activate STAT3, our data implicates that STAT3 plays a vital role in the regulation of autophagy through its contributions to the positive feedback loop between ROS and autophagy. Overall, our findings reveal an important role for STAT3/LC3/ROS in Nx-mediated anti-pancreatic cancer effects.

## INTRODUCTION

Autophagy is a dynamic multistep process in which essential autophagy genes (Atg) participate in forming double-membrane phagophores that engulf damaged cellular proteins, lipids, and organelles before delivering them to lysosomes for subsequent degradation[1-3]. Under normal physiological conditions, autophagic activity is low. However, it can be induced in response to a variety of stimuli including nutrient limitation, oxidative stress, hypoxia, metabolic demands, endoplasmic reticulum stress, physiological agents, inflammatory and immunological signaling to protect cells from stress. Therefore autophagy contributes to cellular homeostasis by removing damaged organelles and maintaining their normal turnover[4, 5]. The role of autophagy in carcinogenesis is complex with reports demonstrating functions in tumor promotion and suppression as well as a contribution to therapeutic resistance[6-8]. For example, curcumin suppresses malignant glioma growth through autophagy induction[9]. Additionally, tamoxifen induces apoptosis in human breast cancer cells through autophagy and endoplasmic reticulum stress[10, 11]. Further, compared to other cancer types such as lung and breast, a higher basal level of autophagy is observed in pancreatic cancer cells and in later stages of pancreatic tumor development[12].

Interestingly, a higher level of autophagy is accompanied by accumulation of reactive oxygen species (ROS) during the development and progression of pancreatic cancer[13, 14]. Many stimuli including nutrient starvation, hypoxia, oxidative stress and some cancer therapy drugs that induce autophagy, also induce ROS. Further, similar to autophagy, both induction and inhibition of ROS promotes cell death in cancer cells including pancreatic by disrupting the redox balance[15-17]. These data suggest a potential crosstalk between autophagy and ROS. However, the role of this crosstalk during pancreatic tumorigenesis is not established. It has been reported that oncogenic Ras-mediated transformation and tumor growth depends on autophagy [18]. Further pancreatic tumors display elevated levels of autophagy [12]. Given the high preponderance of oncogenic Ras mutations, drugs targeting autophagy, ROS and their possible crosstalk may have significant therapeutic potential for successful management of pancreatic cancer [18].

Over 40,000 cases of pancreatic cancer (PanCA) are diagnosed worldwide each year [19]. Only 10-20% of these patients have the opportunity for tumor resection. The majority of patients must be treated with chemotherapy, which can lead to therapeutic resistance and an overall median survival of < 6 months. The lack of effective treatment options, late stage diagnosis and development of therapeutic resistance results in a dismal 5-year survival rate of approximately 5% and this has remained stable for several decades [20, 21]. Much evidence shows that the development of therapeutic resistance is due to elevated levels of oxidative stress in tumor cells and deregulation of multiple tumor cell survival signaling pathways including autophagy [22, 23]. Natural products provide a bountiful source of new chemotherapeutics. Recently we reported that Nexrutine^®^ (Nx), a *Phellodendron amurense* bark extract exhibits excellent anticancer activity in human pancreatic cancer cells through selective modulation of inflammatory signaling via STAT3/NFκB/Cox-2[24]. However, Nx's potential to abrogate autophagy and ROS remains to be determined. Given the apparent anti-inflammatory and anti-proliferative role for Nx, in this study, we investigated the potential of Nx to modulate autophagy, ROS, and possibly their crosstalk. Our results show that the effects of Nx are associated with inhibition of autophagy and decreased intracellular ROS generation. Quenching ROS with N-acetyl-L-cysteine (NAC) inhibited autophagy suggesting that depletion of ROS contributes to Nx-induced inhibition of autophagy. Further, pharmacological inhibition of early-stage (using 3-methyladenine (3-MA), but not late-stage (using chloroquine (CQ) autophagy reduced ROS generation. Thus, suggesting autophagosome formation contributes to Nx-induced reduction of ROS. Remarkably, the combination of Nx with CQ led to enhanced PanCA cell proliferation inhibition with no significant effect on apoptosis. Analysis of these data using isobologram analysis indicated a moderate synergistic to strong additive activity. We also show that genetic and pharmacological inactivation of an inflammatory transcription factor, STAT3, is associated with reduced expression of LC3, which suggests that STAT3 transcriptionally inhibits the LC3 gene. These data provide STAT3/LC3/ROS modulation as a possible mechanism contributing to Nx-induced anti-pancreatic cancer effects. These data support further development of Nx as a promising anticancer agent targeting STAT3/LC3/ROS.

## RESULTS

### Nx modulates autophagy proteins

We previously reported that Nx inhibits proliferation of pancreatic cancer cells [24]. Though the underlying mechanism of Nx's anti-pancreatic cancer effect is still unclear. Recent reports demonstrate the requirement of elevated levels of autophagy for pancreatic tumor growth. This evidence suggests that the inhibition of autophagy may be a potential therapeutic target for PanCA management [12, 18]. Due to this unique characteristic feature and given that both autophagy induction and inhibition could be associated with cell death; we investigated the effect of Nx on autophagy. In consideration of the high preponderance of K-Ras mutations (>90%) in PDAC, we tested the impact of Nx on autophagy using human pancreatic cancer cells that differ in their Ras status [18]. We used mutant K-Ras (Capan-2, AsPC-1, MIAPaCa-2) and wild type K-Ras (BxPC-3) cell lines in this study.

Upon autophagy induction, light chain 3 (LC3, microtubule-associated protein) conjugates to phosphatidylethanolamine to form LC3-II and targets autophagic membranes to form autophagosomes [25, 26, 27]. A cargo protein, p62, in association with LC3-II is incorporated into the autophagosome which then fuses with lysosomes for subsequent degradation. Therefore, autophagic activity is positively and inversely associated with levels of LC3 and p62 respectively. Further, the autophagy gene Atg5 (required for autophagy) plays an essential role in the autophagosomal membrane elongation [28]. Therefore, in addition to examining autophagosome formation using immunofluorescence, we measured the levels of LC3, p62 and Atg5 as markers to monitor autophagy following treatment with Nx [25-28].

**Figure 1:**
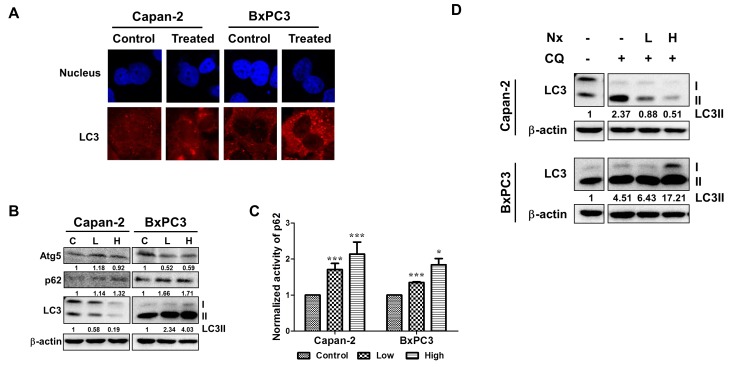
Nx treatment inhibits autophagy in human pancreatic cancer cell lines A. Logarithmically growing human pancreatic cancer cells Capan-2 and BxPC-3 treated with or without Nx (150 μg/ml for Capan-2 and 60 μg/ml for BxPC-3) for 24h were used to determine LC3 levels using immunofluorescence microscopy. Experiment was repeated three times and a representative picture is shown. B. Whole cell extracts prepared from logarithmically growing Capan-2 and BxPC-3 cells treated with low and high dose Nx (doses shown in table 1) for 24h were used in immunoblot analysis with Atg5, p62 and LC3. The membrane was probed with β-actin for loading control. Quantification data normalized to -actin were shown below the blot. Representative blot from multiple experiments is shown. C. Logarithmically growing Capan-2 and BxPC-3 cells treated with low and high Nx for 24h were used to determine the levels of p62 using ELISA (Enzo life sciences, NY). Statistical significance between groups was determined using students t-test and p values less than 0.05 was considered significant (* p<0.05, and *** p<0.005). D. Whole cell extracts prepared from logarithmically growing Capan-2 and BxPC-3 cells treated with different doses of Nx (low and high) in the presence and absence of autophagy inhibitors (25μM CQ) for 24h were used in immunoblot analysis with LC3 antibody. Quantification data normalized to β-actin were shown below the blot. Representative blot from multiple experiments is shown.

As shown in figure 1a, following treatment with Nx for 24h, we observed increased formation of puncta in both Capan-2 and BxPC-3 indicating the formation of autophagosomes irrespective of oncogenic Ras mutation. Similar results were obtained with AsPC-1 and MIAPaCa-2 cells (supplementary figure 1). It should be mentioned that in MIAPaCa-2 cells, we also observed diffuse cytoplasmic staining for LC3 in addition to the formation of puncta (supplementary figure 1). When we measured the levels of Atg5, LC3 and p62 under these experimental conditions, basal levels of Atg5, LC3 and lipidated membrane-associated LC3-II was detectable in both Capan-2 and BxPC-3 cells (figure 1b). However, it should be mentioned that basal levels of Atg5 and LC3II were relatively higher in BxPC-3 compared to Capan-2 cells (figure 1b). Treatment with Nx showed decreased Atg 5 (8%) and LC3II (81%) as well as increased p62 (32%) in Capan-2 cells (figure 1b). On the other hand, BxPC-3 cells demonstrated a more pronounced Atg5 decrease (41%) and p62 increase (71%) with increased levels of LC3II (400%). It is noteworthy to mention that LC3II can be generated in an autophagy-independent manner as suppression of Beclin1 with shRNA or Beclin1-deifienct embryonic fibroblasts do not form autophagosomes [29, 30]. In addition, we measured p62 levels using an independent ELISA-based approach and obtained similar results (figure 1c). The observed autophagosome formation could be due to increased accumulation or a block in the later steps of autophagy (degradation of lysosomal proteins) leading to inhibition or activation of autophagy [31, 32].

**Table 1:** Doses used in experiments

Cell line	Nx(μg/ml)		
	Low	Medium	High
Capan-2	50	100	150
BxPC3	20	40	60
AsPC-1	50	100	150
MIA PaCa-2	20	40	50

### Nx inhibits autophagy

In order to determine whether the observed modulation of autophagic activity leads to inhibition or activation of autophagy, we measured autophagic flux. This was accomplished by measuring the levels of LC3-II protein in the presence and absence of pharmacological inhibition of autophagy with CQ, inhibitor of autolysosome formation. As shown in figure 1d, incubation with CQ led to ~2.3 and 4.5-fold increase in the levels of LC3-II in Capan-2 and BxPC-3 cells respectively as compared to levels without CQ, indicating increased autophagic flux. However, the observed CQ-induced increased levels of LC3-II decrease to ~0.5-fold (78% decrease) in the presence of Nx for Capan-2 and increase to 17-fold (380% increase) in BxPC-3 cells. These data suggest that LC3-II is not delivered to lysosome in the presence of Nx for degradation, which results in decreased autophagy in Capan-2 cells. However, in BxPC-3 cells we see increased levels of LC3-II. Although other possibilities exist, we interpret our findings to suggest that Nx increases levels of LC3-II possibly in an autophagy-independent manner [29, 30]. Despite these differences in LC3-II, we observed increased levels of p62 in both Capan-2 and BxPC-3 cells (figure 1b). Taken together; these data suggest that there is an increase in the basal level of autophagy due to blockage of LC3-II turnover and a Nx-mediated decrease in autophagic flux.

### Nx enhances proliferation inhibition in combination with autophagy inhibitors

**Figure 2:**
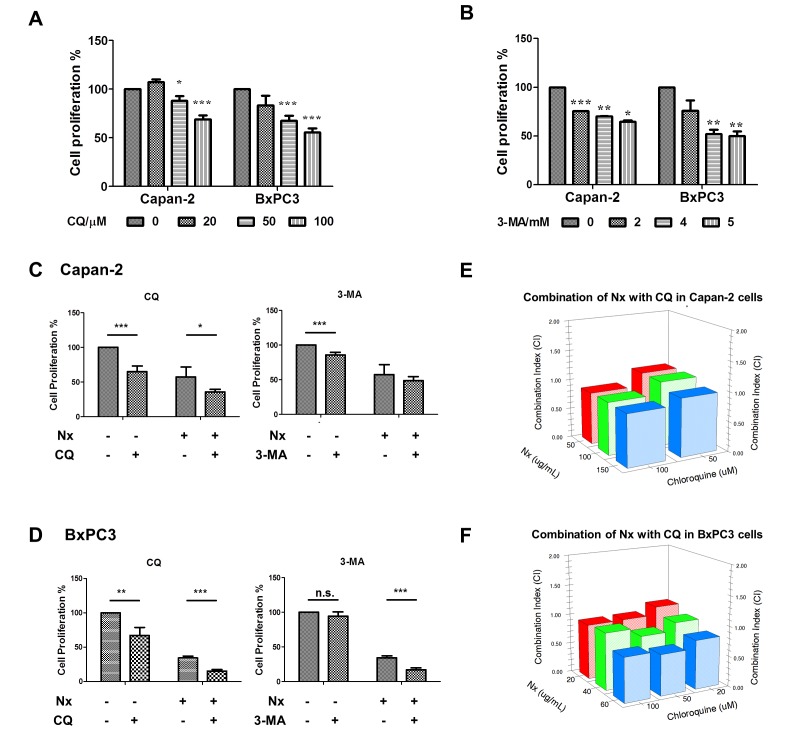
Enhanced antiproliferative effects of Nx in combination with autophagy inhibitors A and B. Logarithmically growing Capan-2 and BxPC-3 cells treated with increasing doses of CQ (25μM; panel A) or 3-MA (1mM; panel B). Following 24h incubation, cell proliferation was measured using Cell Titer 96 Aqueous One solution assay. Percent cell proliferation in cells treated with compounds was calculated with respect to solvent control and data was presented as average+sd. Statistical significance between groups was determined using students t-test and p values less than 0.05 was considered significant (* p<0.05, ** p<0.01, and *** p<0.005). Capan-2 (panel C) and BxPC-3 (panel D) cells were treated concurrently with Nx and CQ (25μM) or 3-MA (1mM) for 24h. Following this incubation, cell proliferation was measured as described for panels A and B. Capan-2 (panel E) and BxPC-3 (panel F) cells were treated concurrently with increasing doses of Nx and CQ for 24h and cell proliferation was measured as described above. Combination indices were calculated using isobologram analysis.

We next assessed the impact of Nx in combination with CQ and 3-MA on proliferation inhibition. First, proliferation of Capan-2 and BxPC-3 cells was measured following treatment with different doses of CQ (20-100 μM) and 3-MA (2-5 mM) for 24h. As a positive control, we treated cells with different doses of rapamycin for 24h. As shown in figure 2a and b, both autophagy inhibitors significantly inhibited proliferation of these cells in a dose-dependent manner. Both cell types showed no significant change in proliferation when treated with rapamycin (supplementary figure 2a). We then assessed the influence of pharmacological inhibition of autophagy on the anti-proliferative effects of Nx by pre or concurrent treatment with autophagy inhibitors (CQ, 25μM; 3-MA, 1mM). Pretreatment with autophagy inhibitors did not influence the anti-proliferative effects of Nx (supplementary data figure 2b). Both CQ and 3-MA enhanced proliferation inhibition when combined with Nx in BxPC-3 cells. However, only CQ produced a significant increase in proliferation inhibition when combined with Nx in Capan-2 cells (figure 2 c and d). Further, analysis of these data using isobologram analysis indicated that Nx, in combination with CQ, showed strong additive to moderate synergistic activity in both cell types (figure 2e and f). No growth inhibitory effects were observed in MIAPaCa-2 or AsPC-1 cells after treatment with Nx in combination with CQ or 3-MA (supplementary figure 2 c and d). Taken together these results suggest that combination of autophagy inhibitors with Nx could have additive to moderate synergistic effects depending on the cell type.

It is reported that inhibition of autophagy can synergize with other agents to facilitate apoptosis [33, 34]. We investigated this possibility by testing the ability of Nx to modulate apoptosis in the presence of pharmacological inhibition of autophagy. Treatment with autophagy inhibitors alone or in combination with Nx showed no statistically significant change in apoptosis under our experimental conditions (supplementary figure 3a). Although these data suggest that combination of Nx and autophagy inhibitors may exert enhanced anti-cancer effects, the underlying biological process is unclear. Since cells could die through other processes besides apoptosis, it is possible that combination of Nx and autophagy inhibitors could trigger one of these processes (such as necroptosis). In addition, recent reports suggest (i) that autophagy promotes the expression of markers associated with epithelial mesenchymal transition (EMT) and invasion in hepatocellular carcinoma cells and (ii) the association of autophagy inhibition with decreased pulmonary metastasis in a hepatocellular carcinoma model [35, 36]. However, it is not clear whether Nx, in combination with autophagy inhibitors, modulates markers associated with EMT and metastasis *in vivo.*

### Nx treatment inhibits intracellular ROS production

**Figure 3:**
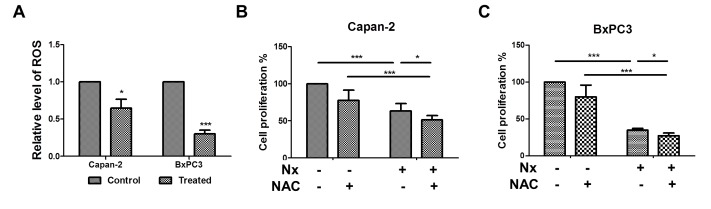
Nx reduces intracellular ROS generation A. Logarithmically growing Capan-2 and BxPC-3 cells were treated with 150 μg/ml and 60 μg/ml Nx respectively for 24h. Generation of intracellular ROS was determined by CellROX^®^ deep red reagent (Invitrogen, NY) following manufacturer's instructions. Fold change in intracellular ROS production was calculated relative to untreated samples.B and C. Logarithmically growing Capan-2 (B) and BxPC-3 (C) cells were treated with 150 μg/ml and 60 μg/ml Nx respectively in the presence of N-acetyl cysteine, NAC (5 mM) for 24h. Cell proliferation was measured using the Cell Titer 96 Aqueous One solution assay. Percent cell proliferation in cells treated with compounds was calculated with respect to solvent control and data was presented as average+sd. Statistical significance between groups was determined using students t-test and p values less than 0.05 was considered significant (* p<0.05, and *** p<0.005).

Elevated ROS accompanies pancreatic tumorigenesis[12]. Therefore, we determined the effect of Nx on ROS generation. Intracellular ROS was measured using CellROX^®^ Deep Red Reagent (Carlsbad, CA) by flow cytometry. We found that treatment with Nx significantly inhibited ROS levels in Capan-2 and BxPC-3 cells (figure 3a). Under these conditions, we observed that BxPC-3 cells were more sensitive than Capan-2 cells. Similar results were obtained in AsPC-1; however, ROS production was increased in MIAPaCa-2 cells (supplementary figure 3b). In order to determine whether ROS contributes to Nx-induced anti-proliferative effects, endogenous ROS levels were quenched by (i) pretreating cells with NAC followed by treatment with Nx or (ii) concurrently in the presence of Nx. Quenching ROS demonstrated a marginal, but statistically significant decrease in Nx-induced proliferation inhibition of Capan-2 and BxPC-3 cells (figure 3b and 3c). Similar results were obtained in AsPC-1 and MIAPaCa-2 cells (supplementary figure 3c). These data suggest that Nx alone effectively quenches ROS and that ROS may not play a direct role in mediating Nx-induced anti-proliferative effects. However, whether the observed Nx-mediated ROS reduction contributes to autophagy inhibition is not clear.

### Pharmacological approaches to decipher Nx-mediated modulation of crosstalk between ROS and autophagy

**Figure 4:**
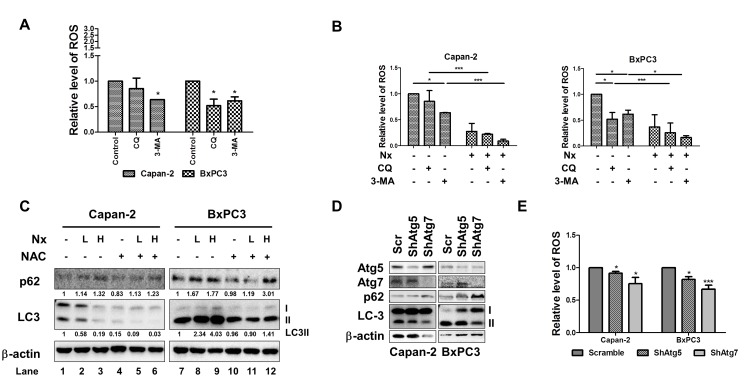
Crosstalk between ROS and autophagy A. Logarithmically growing Capan-2 and BxPC-3 cells were treated with autophagy inhibitors (25μM CQ or 1mM 3-MA) for 24h. Level of intracellular ROS was determined by CellROX^®^ deep red reagent (Invitrogen, NY) following manufacturer's instructions. Fold change in intracellular ROS was calculated relative to untreated samples. Statistical significance between groups was determined using students t-test and p values less than 0.05 was considered significant. B. Capan-2 and BxPC-3 cells were treated with Nx as described in legends figure 2 in the presence or absence of autophagy inhibitors (25μM CQ or 1mM 3-MA) for 24h for measuring intracellular levels of ROS. Level of intracellular ROS was determined by CellROX^®^ deep red reagent (Invitrogen, NY) following manufacturer's instructions. Fold change in intracellular ROS was calculated relative to untreated samples. Statistical significance between groups was determined using students t-test and p values less than 0.05 was considered significant with *indicating p<0.05; **; p<0.01; and *** p<0.005). C. Protein levels of p62 and LC3 in Capan-2 and BxPC-3 cells treated with Nx in the presence and absence of 5 mM NAC for 24h were determined by immunoblot analysis. Quantification data normalized to β-actin were shown below the blot. Representative blot from multiple experiments is shown. D. Effect of knocking down Atg5 and 7 on levels of p62 and LC3 and intracellular ROS in Capan-2 and BxPC-3 cells: Levels of Atg5, 7, p62 and LC3 were determined by immunoblot analysis of Capan-2 or BxPC-3 cells stably expressing Atg5 or Atg7 shRNA. E. Intracellular ROS production was measured in Capan-2 or BxPC-3 cells stably expressing Atg5 or Atg7-specific shRNA or scrambled shRNA using CellROX^®^ deep red reagent (Invitrogen, Carlsbad, CA; E). Statistical significance between groups was determined using students t-test and p values less than 0.05 was considered significant (*p<0.05 and ***p<0.005).

To explore the potential crosstalk between ROS and autophagy, we measured (i) autophagy by quenching endogenous ROS levels with NAC or (ii) ROS levels in the presence of autophagy inhibitors. As shown in figure 4a, pharmacological inhibition of early stage autophagy reduced ROS levels in Capan-2 and BxPC-3 cells. However, pharmacological inhibition of late stage autophagy reduced ROS levels in BxPC-3, but not in Capan-2 cells. Similarly, pharmacological inhibition of autophagy reduced ROS levels in AsPC-1 cells (supplementary figure 4a). Interestingly, both pharmacological inhibition of autophagy and Nx induced ROS levels in MIAPaCa-2 cells.

We then tested whether autophagy is required for Nx-mediated ROS inhibition. As shown in figure 4b and supplementary figure 4b, pharmacological inhibition of autophagy with 3-MA or CQ reduced ROS levels, although no statistically significant effect was observed on Nx-mediated ROS reduction. We next conducted converse studies by analyzing the levels of LC3 and p62 in the presence of NAC. As shown in figure 4c, quenching ROS significantly reduced levels of LC3-II in Capan-2 (compare lanes 1 and 4) with no significant effect in BxPC-3 cells (compare lanes 7 and 10). Moreover, the observed decrease in LC3-II levels was further reduced in the presence of Nx for Capan-2 (compare lanes 4 to 5 and 6) but not in BxPC-3 cells (compare lanes 10 to 11 and 12). It should be mentioned that quenching ROS also marginally decreased levels of p62 in both Capan-2 and BxPC-3 cells; however, treatment with Nx rescued the observed decrease (figure 4c). Although quenching ROS reduced LC3 levels, treatment with Nx had no further effect in AsPC-1 cells (supplementary figure4c). Taken together our results demonstrate that inhibition of autophagy decreased ROS levels and that quenching ROS (with NAC or Nx) inhibits autophagy, thereby suggesting a positive feedback loop. To further validate the crosstalk between autophagy and ROS, we generated Atg5 and Atg7 stable knockdown cell lines using Capan-2 and BxPC-3. As shown in figure 4d, knock down of Atg5 or Atg7 resulted in reduced LC3-II and increased p62 protein levels for both Capan-2 and BxPC-3 cells compared to control cells transduced with scramble shRNA. This indicates that autophagy activation was inhibited.

We examined the effects of Atg5 and Atg7 silencing on ROS levels, and found that silencing either Atg5 or Atg7 caused a marginal, but statistically significant inhibition of ROS levels in both cell lines. The reduction was more significant in BxPC-3 cells with Atg7 knockdown (figure 4e). This data was consistent with our results indicating a potential positive feedback loop between autophagy and ROS. Overall, our data implicates that ROS contributes to autophagy and vice versa in these cells and that Nx inhibits both autophagy and levels of ROS. However the mechanism through which this positive feedback loop is inhibited is unclear.

### STAT3 regulates autophagy

**Figure 5:**
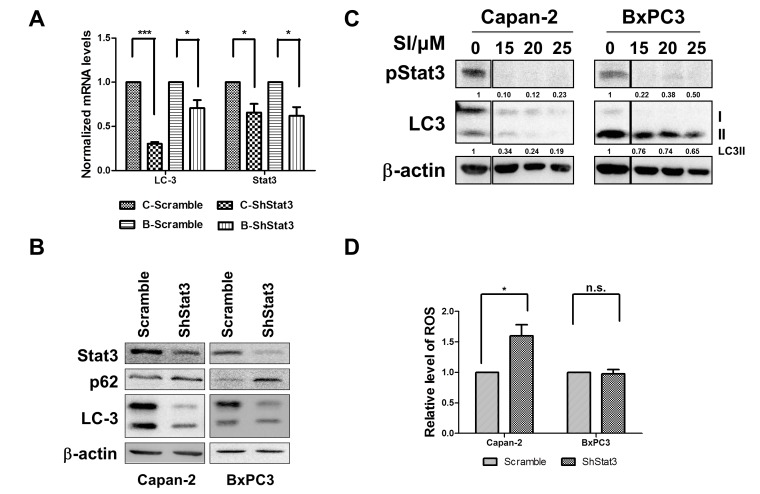
STAT3 is a modulator of autophagy A and B. Effect of stable knock down of STAT3 on expression and levels of LC3 and Stat3 in Capan-2 and BxPC-3 cells. Expression of mRNA and protein levels of p62 and LC3 were determined by RT-PCR and western blot analysis of Capan-2 or BxPC-3 cells stably expressing STAT3 or scrambled shRNA. C. Capan-2 and BxPC-3 cells were treated with different concentrations of STAT3 inhibitor (SI; Sttatic, Santa Cruz, CA) for 24h. Levels of p STAT3 and LC3 were determined by immunoblot analysis. Quantification data normalized to β-actin were shown below the blot. Representative blot from multiple experiments is shown. D. Intracellular ROS was measured in Capan-2 or BxPC-3 cells stably expressing STAT3-specific shRNA or scrambled ShRNA using CellROX^®^ deep red reagent (Invitrogen, Carlsbad, CA; D). Statistical significance between groups was determined using students t-test and p values less than 0.05 was considered significant (* p<0.05).

**Figure 6:**
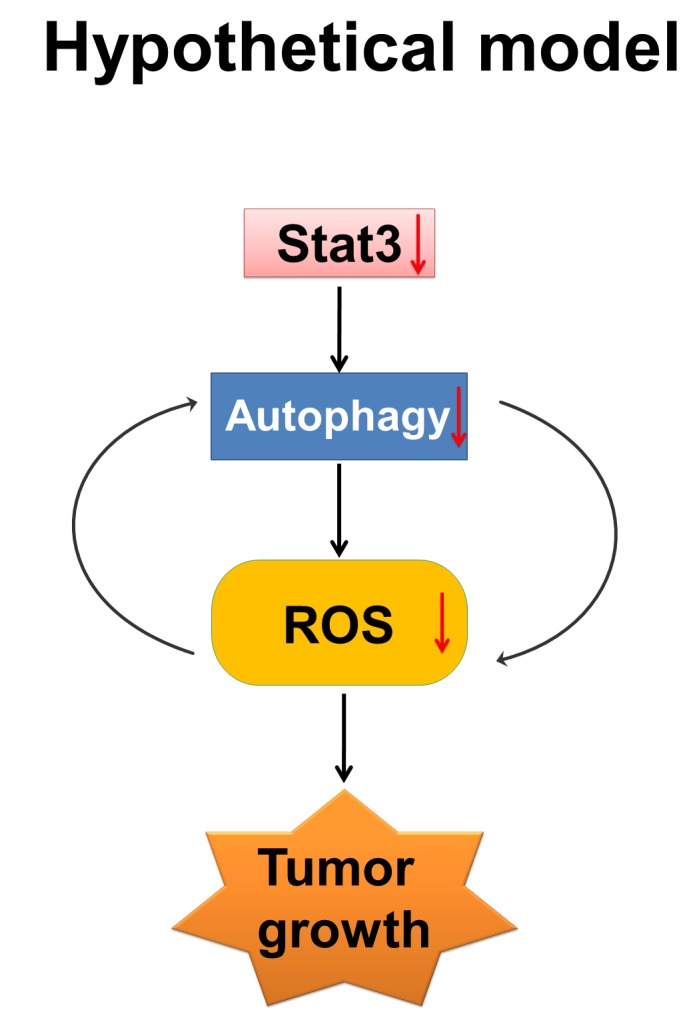
Hypothetical model ROS, autophagy and inflammation play critical roles in PanCA progression. ROS is known to activate inflammatory transcription factor STAT3. Our data indicates that (i) STAT3 regulates autophagy possibly by transcriptional regulation of LC3; and (ii) treatment with Nx inhibits autophagy and reduces ROS generation. Further, pharmacological inhibition of ROS inhibits autophagy while pharmacological or genetic inhibition of autophagy inhibits ROS suggesting a positive feedback. Our working model is that Nx inhibits pancreatic cancer cell growth by interrupting the positive feedback loop between autophagy, ROS and STAT3. Nx mediated inhibition is indicated by downward arrow.

In previous studies from our laboratory, we found a marked decrease in the levels of DNA binding activity by the active and inactive forms of STAT3, an inflammatory mediator, which was associated with proliferation inhibition in pancreatic cancer cells after treatment with Nx [24]. STAT3 signaling plays an important role, not only in tumorigenesis but also in the development of therapeutic resistance [37-39]. Constitutive activation of STAT3 has been reported in PanCA patients [40]. Recently, it was reported that silencing receptor for advanced glycation end products (RAGE) or Atg5 using shRNA significantly inhibited IL-6 induced autophagy and mitochondrial STAT3 activation in pancreatic cancer cells [41]. These data suggest the requirement of autophagy for activation of IL-6- STAT3 signaling in pancreatic carcinogenesis through RAGE [41]. In order to determine whether the observed Nx-mediated inhibition of autophagy is related to decreased activation of STAT3, we examined the expression and protein level of LC3 and p62 in STAT3 stable knockdown cell lines. We found that STAT3 knockdown significantly reduced LC3 mRNA expression and protein levels compared to scrambled control cells (figure 5a and b). These data suggest that STAT3 transcriptionally inhibits the expression of LC3. Further, protein levels of p62 increased under these conditions implying inhibition of autophagy (figure 5b). Additionally, pharmacological inhibition of STAT3 activation using STAT3 inhibitor V (Stattic, Santa Cruz, CA) showed a dose-dependent reduction in pSTAT3 and LC3-II in these cells (figure 5c). These data suggest the distinct possibility that STAT3 transcriptionally regulates autophagy through LC3. Surprisingly, significantly elevated levels of intracellular ROS were detected in Capan-2 STAT3 knockdowns with no significant change in BxPC-3 cells as compared to scrambled controls (figure 5d). Taken together these results show that STAT3 transcriptionally regulates LC3 and that autophagy induces ROS. Since our data demonstrates reduced STAT3 inactivation with Nx treatment, we believe that Nx (i) inhibits autophagy by reducing STAT3-mediated activation of autophagy (LC3; see hypothetical model in figure 6).

## DISCUSSION

The near equal rates of incidence and mortality make pancreatic cancer one of the most aggressive human malignancies worldwide[12, 42]. Late stage diagnosis, lack of prognostic markers, and therapeutic resistance are major challenges for effective clinical management of PanCA. Evidence suggests chronic inflammation associated with activation of inflammatory mediators including STAT3, NFκB, elevated ROS levels, and enhanced autophagy accompany pancreatic tumorigenesis [39].

We discovered the promising anti-cancerous activity for Nexrutine® (Nx), a *Phellodendron amurense* bark extract, using both *in vitro* and *in vivo* prostate cancer models [43]. These studies demonstrated the unique ability of Nx to inhibit multiple deregulated signaling pathways in cancer cells leading to tumor growth suppression [43-45]. These published studies also observed no significant change in the body weight of animals receiving Nx, which is indicative of its non-toxic nature. Since then, its anti-cancerous activity has been confirmed in other tumor models including DMBA-TPA induced epidermal carcinogenesis, breast, and melanoma [44-47]. In addition, we recently demonstrated the ability of Nx to reduce fibrosis in a pancreatic cancer model which may possibly occur through modulation of STAT3/NFκB/EP4 axis [24]. Collectively, these published studies suggest that Nx is a promising, over the counter, herbal supplement with anti-cancerous activities. However, in order to develop Nx for clinical use, the underlying molecular mechanism should be deciphered. Accordingly, in this study, we explored whether the anti-proliferative effects of Nx were mediated via regulation of ROS and autophagy.

Consistent with published studies, we observed high basal levels of autophagy in human pancreatic cancer cells [12, 39]. Using multiple biochemical approaches and pharmacological inhibitors of autophagy we show that Nx inhibits autophagy. Treatment with Nx reduced intracellular production of ROS in these cell lines irrespective of oncogenic Ras status. It is noteworthy to mention that the MIAPaCa-2 cell line with oncogenic K-Ras mutation shows increased ROS production in response to Nx. The reason for this increase is not apparent at this time. Nevertheless, we believe that this could be related to the poorly differentiated carcinoma status of this cell line as opposed to adenocarcinoma in other cell types used in the study [48]. Interestingly, production of ROS has been reported as a stimulus for autophagy induction [49, 50]. Further, ROS activation is associated with tumor growth suppression [51, 52]. These data indicate a positive association between ROS and autophagy and suggest a potential link between ROS and autophagy in mediating Nx's anti-proliferative effects. Consequently, we tested the crosstalk between ROS and autophagy using pharmacological and genetic approaches. Pharmacologic (via 3-MA) and genetic inhibition of autophagy resulted in decreased ROS generation in Capan-2, AsPC-1 and BxPC-3 cells, suggesting a positive feedback loop between ROS and autophagy. Additionally, enhanced ROS inhibition was observed when Nx was combined with 3-MA which further demonstrated that autophagosome formation is one of the targets of Nx. CQ, which inhibits autolysosome formation, reduced ROS generation only in BxPC-3 cells but not in Capan-2 cells suggesting a possible differential crosstalk between autophagy and ROS that may be dependent on the status of oncogenic Ras. However, we found quenching ROS by NAC did not impact Nx-mediated autophagy. This implies that despite the positive interaction between ROS and autophagy, Nx can inhibit ROS and autophagy independently.

It is well established that autophagy and ROS can facilitate cell death or cell survival depending on the nature of stimuli and cellular context [6, 53]. Given our observation of reduced intracellular ROS levels upon autophagy inhibition, we tested the impact of autophagy on Nx induced anti-proliferative activities. Pharmacologic inhibition of autophagy decreased cell proliferation and was further enhanced in the presence of Nx. Interestingly; the observed increase in proliferation inhibition was not associated with apoptosis induction. These observations are in contrast with some published studies, which show an association between autophagy inhibition and enhanced apoptosis [33, 53]. On the other hand, our results suggest that autophagy inhibition synergizes with Nx (to prevent autophagy activation) resulting in enhanced proliferation inhibition rather than apoptosis activation. We do not know whether prolonged proliferation inhibition can lead to cell death through other mechanisms including necrosis or necroptosis [54]. This requires further investigation. In addition, recent reports suggest that (i) autophagy promoted the expression of markers associated with epithelial mesenchymal transition (EMT) and invasion in hepatocellular carcinoma cells and (ii) association of autophagy inhibition with decreased pulmonary metastasis in a hepatocellular carcinoma model [35, 36]. However, it is not clear whether Nx in combination with autophagy inhibitors modulate markers associated with EMT and metastasis *in vivo.*

Our studies here divulge STAT3 as an important link between oxidative stress and autophagy. We found for the first time that silencing STAT3, either genetically or pharmacologically, resulted in reduced expression of LC3 in Capan-2 cells suggesting that STAT3 transcriptionally regulates LC3. Further, silencing STAT3 increased levels of p62 suggesting STAT3 positively regulates autophagy activation. Remarkably, silencing STAT3 also resulted in increased intracellular production of ROS under these experimental conditions in Capan-2 cells. Overexpression and activation of STAT3 occurs in many human cancers including pancreatic cancer and leads to tumor cell growth, invasion, and metastasis [36, 37]. ROS is known to activate various transcription factors including STAT3 to regulate wide variety of cellular processes including inflammation, cell transformation, tumor cell survival, proliferation, invasion, angiogenesis, and metastasis [55-57]. Moreover, ROS induces the transcriptional activity of STAT3 in autophagy activated cancer cells [57]. We recently reported that Nx-induced anti-proliferative effects are mediated through deactivation of STAT3 signaling [24]. Based on these published data we suggest that STAT3 may play a vital role in (i) autophagy by transcriptionally regulating LC3 and (ii) inflammatory signaling via ROS. Treatment with Nx inhibits STAT3 activation leading to transcriptional down regulation of LC3 with consequent autophagy inhibition. Therefore, STAT3 might play a key role as a link between inflammation and autophagy possibly via oxidative stress. Targeting inflammatory mediators such as STAT3, ROS, and autophagy may act as an effective and viable strategy for pancreatic cancer management.

It is well established that more than 90% pancreatic tumors display K-Ras mutation, which contributes to therapeutic resistance [58-60]. Therefore our findings demonstrating the responsiveness of cells with and without oncogenic Ras mutation to Nx-induced biological effects is significant. Secondly, use of Nx has several advantages including: (i) Nx's selectiveness towards actively proliferating cells with no toxicity in animal models [44, 46, 47]; and (ii) Nx's ability to target multiple signaling pathways which are often deregulated in tumors due to its inherent complexity as an herbal extract. Moreover we also found that CQ can enhance the anti-proliferative activity of Nx additively. CQ is an established antimalarial drug and is currently being used in combination with various chemotherapeutic agents in clinical trials including pancreatic cancer [12, 61] Further, it is important to emphasize that Nx is currently undergoing clinical trials in prostate cancer patients. These initial findings indicate it is safe and well tolerated, thus signifying the translational potential of Nx. These data strongly support its use and warrant studies to test this potentially non-toxic novel combination of agents in preclinical studies as an effective strategy for PanCA management.

## MATERIALS AND METHODS

### Cell lines and Chemicals

Human pancreatic cancer cell lines Capan-2, MIAPaCa-2 and AsPC-1 (with K-Ras mutation) and BxPC-3 (wild type K-Ras) were obtained from American Type Cell Culture (ATCC, Rockville, MD). Capan-2 and BxPC-3 cells with STAT3 stably knocked down were generous gifts from Dr. James Freeman (The University of Texas Health Science Center at San Antonio, TX). All cell lines except MIAPaCa-2 were grown in RPMI medium (Mediatech, Inc., Manassas, VA) supplemented with 10% fetal bovine serum, 100 μg/ml penicillin-streptomycin, and 100 μg/ml Amphotericin in a humidified incubator at 37°C and 5% CO_2_. MIAPaCa-2 cells were grown in DMEM medium supplemented with high glucose, 10% fetal bovine serum plus 5% horse serum. Stock solutions of Nx (5 mg/ml) were prepared by dissolving powdered Nx in 50% dimethyl sulfoxide. Doses of Nx used in the study are shown in table 1. Monoclonal antibodies (STAT3, pSTAT3) and STAT3 inhibitor V were purchased from Santa Cruz Biotechnology (Santa Cruz, CA). Monoclonal antibodies for LC-3 and Atg5 (Enzo life Science; Farmingdale, NY) and p62 were purchased from Cell Signaling Technologies (Beverly, MA).

### Biochemical experiments

Cell proliferation was measured using CellTiter 96 Aqueous One solution assay (Promega Corporation, Madison, WI) according to the manufacturer's directions as described previously [44, 47]. Cellular apoptosis was measured using APC-Annexin-V assay (BD Biosciences, San Diego, CA). Levels of p62 were measured using p62 ELISA kit according to the manufacturer's instructions (Enzo life Science, Farmingdale, NY). Immunoblot analysis and Real-Time PCR were conducted as previously described [24, 44, 47]. Primers used in real-time PCR are shown in table 2.

**Table 2:** Primers used in real-time PCR

Gene	Forward	Reverse
P62	TGCCCTCCATGTGTAAGA	CAGAAAGTGTCAGAACCAGAG
Atg5	TGGACAGTTGCACCACTAGGAGA	GCAGAGGTGTTTCCAACATTGGCT
Atg7	CCAAGGTCAAAGGACGAAGATA	CCTGGTGTCCATCAATAGGAAG
LC3	TGTCCGACTTATTCGAGAGCAGCA	TTACACCAACAGGAAGAAGGCCTGA
Stat3	TCCAGTCCGTGGAACCATACACAA	AATGCCTCCTCCTTGGGAATGTCA

### Confocal microscopy

Confocal microscopy was performed using LC3 was performed as previously described [24].

### Cytoplasmic ROS detection

Following treatment with different concentrations of Nx, indicated cells were incubated with 5 μm CellROX^®^ Deep Red reagent (Invitrogen, Carlsbad, CA) for 30 min at 37°C. ROS levels were measured by flow cytometry according to the manufacturer's protocol and values reported as mean fluorescence intensity.

### Autophagy Detection

Autophagic vacuole formation was detected using Cyto-ID autophagy detection kit according to the manufacturer's instructions (Enzo life Science, Farmingdale, NY). Autophagosome formation was determined by flow cytometry and values reported as mean fluorescence intensity [62].

### Combination Index analysis

Capan-2 or BxPC-3 cells were treated with Nx and CQ alone and in combination over a range of concentrations for 24h. Following this incubation, cell proliferation was measured. Interaction between Nx and CQ was calculated using combination index (CI) according to Chou and Talalay [63]. CI value of 1 indicates additive and values lower than 1 are indicative of strong synergistic activity.

### Statistical analysis

All experiments were conducted at least three times and the data presented as average ± sd. In order to compare treatment effects, data was normalized to untreated solvent control. Statistical significance among different treatments was determined by ANOVA followed by t-test and p values <0.05 was considered significant.

## SUPPLEMENTARY FIGURES


