# Supplementary material for: STAT3 down regulates LC3 to inhibit autophagy and pancreatic cancer cell growth
Source: Oncotarget. 2014 Mar 15;5(9):2529–41. doi: 10.18632/oncotarget.1810 (PMC4058024; doi:10.18632/oncotarget.1810)
Supplement: Supplementary file 1 [file oncotarget-05-2529-s001.pdf]

# STAT3 down regulates LC3 to inhibit autophagy and pancreatic cancer cell growth – Gong et al

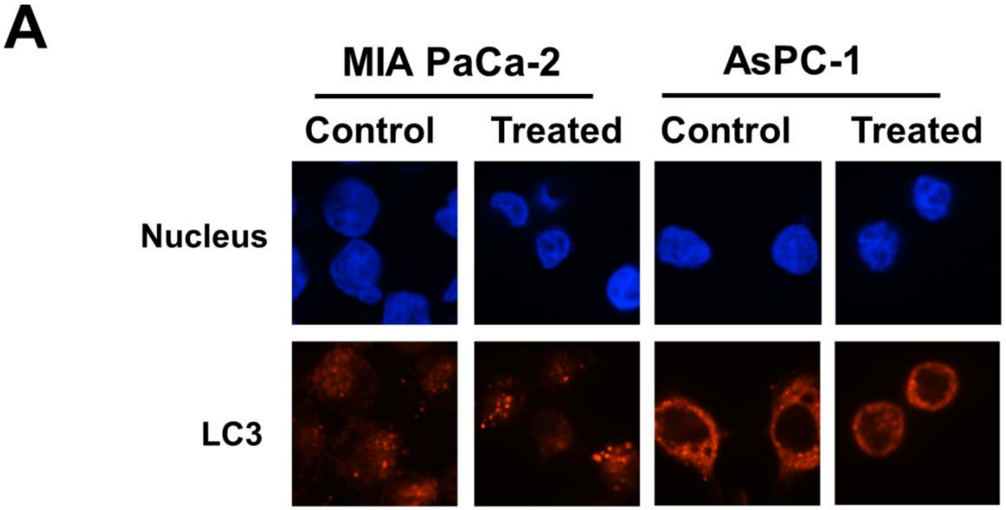


**SF1: Nx treatment inhibits autophagy in human pancreatic cancer cell lines.** Logarithmically growing human pancreatic cancer cells MIAPaCa-2 and AsPC-1 treated with or without Nx (50 µg/ml for MIAPaCa-2 and 150 µg/ml for and AsPC-1) for 24h were used to determine LC3 levels using immunofluorescence microscopy. Experiment was repeated three times and a representative picture is shown.

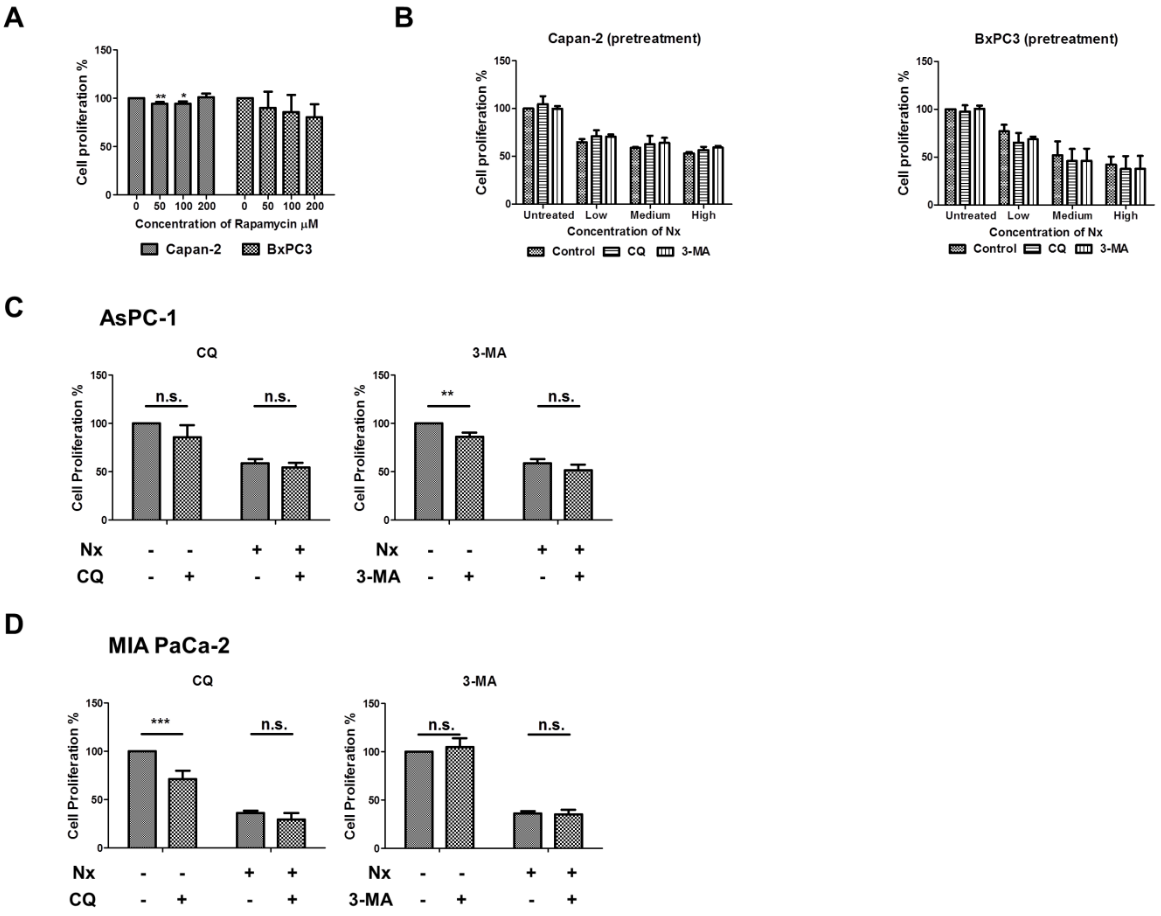


**SF2: Cell proliferation in pancreatic cancer cells: effect of rapamycin and autophagy inhibitors.**
Logarithmically growing human pancreatic cancer cells **(A)** Capan-2 and BxPC-3 cells treated with indicated doses of rapamycin; **(B)** Capan-2 and BxPC-3 were pretreated for 2h with indicated doses of CQ alone (25µM) or 3-MA (1mM) followed by different doses of Nx; **(C)** AsPC-1 and MIAPaCa-2 **(D)** were concurrently treated with Nx in the presence of CQ (25µM) or 3-MA (1mM) for 24h. Cell proliferation measured 24 h after treatment using the Cell Titer 96 Aqueous One solution assay. Statistical significance between groups was determined using students t-test and p values less than 0.05 was considered significant (* p<0.05, **; p<0.01, and *** p<0.005).

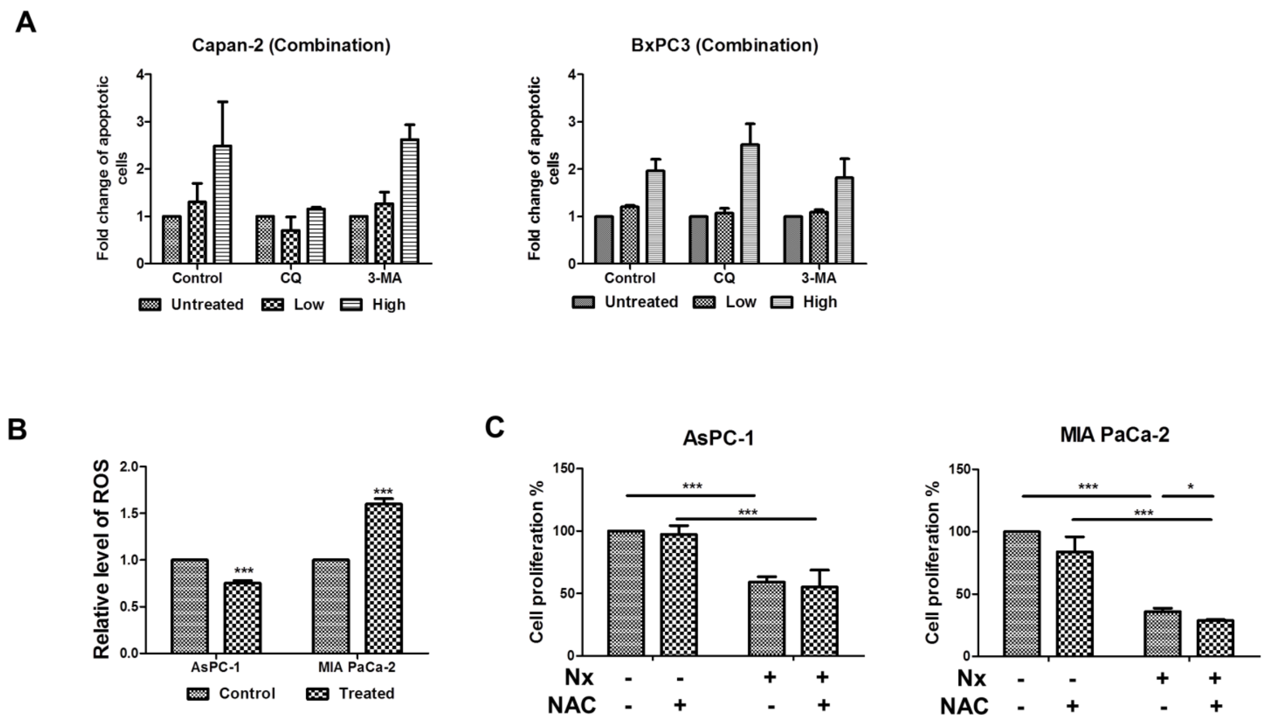


**SF3: Nx in combination with autophagy inhibitors had no effect on apoptosis. A.** Logarithmically growing Capan-2 and BxPC-3 cells treated with increasing doses of Nx alone in combination with CQ (25µM) or 3-MA (1mM) for 24h. Following incubation, apoptosis was determined using APC-Annexin-V assay (BD Biosciences, San Diego, CA). Fold change in apoptotic cells (Annexin positive cells) was calculated by normalizing to solvent treated cells.

**B and C.** Logarithmically growing AsPC-1 and MIAPaCa-2 cells were treated with 150 µg/ml and 50 µg/ml Nx respectively in the absence or presence of N-acetyl cysteine, (NAC 5 mM) for 24h. Generation of intracellular ROS was determined by CellROX® deep red reagent (Invitrogen, NY) following manufacturer's instructions **(B)**. Fold change in intracellular ROS production was calculated relative to untreated samples. Under parallel experimental conditions, cell proliferation was also measured using the Cell Titer 96 Aqueous One solution assay **(C)**.

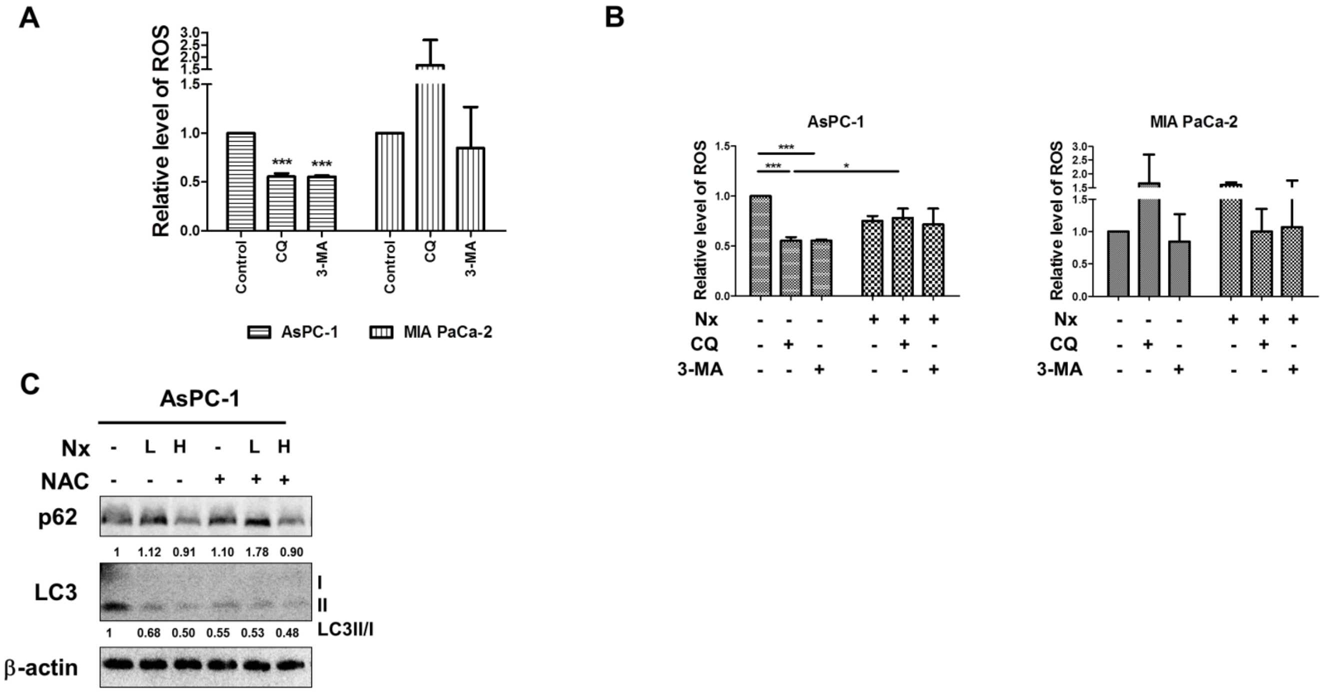


**SF4: Crosstalk between ROS and autophagy: A.** Logarithmically growing AsPC-1 and MIAPaCa-2 cells were treated with autophagy inhibitors (25µM CQ or 1mM 3-MA) for 24h. Level of intracellular ROS was determined by CellROX® deep red reagent (Invitrogen, NY) following manufacturer's instructions. Fold change in intracellular ROS was calculated relative to untreated samples.

**B.** AsPC-1 and MIAPaCa-2 cells were treated with Nx 150 µg/ml and 50 µg/ml Nx respectively in the presence or absence of autophagy inhibitors (25µM CQ or 1mM 3-MA) for 24h for measuring intracellular levels of ROS. Level of intracellular ROS was determined by CellROX® deep red reagent (Invitrogen, NY) following manufacturer's instructions. Fold change in intracellular ROS was calculated relative to untreated samples. Statistical significance between groups was determined using students t-test and p values less than 0.05 was considered significant with * indicating $p<0.05$; and *** $p<0.005$).

**C.** Protein levels of p62 and LC3 in AsPC-1 cells treated with Nx in the presence and absence of 5mM NAC for 24h were determined by immunoblot analysis. Quantification data normalized to β-actin were shown below the blot. Representative blot from multiple experiments is shown.
